# Patient-Centered Pain Care Using Artificial Intelligence and Mobile Health Tools: Protocol for a Randomized Study Funded by the US Department of Veterans Affairs Health Services Research and Development Program

**DOI:** 10.2196/resprot.4995

**Published:** 2016-04-07

**Authors:** John D Piette, Sarah L Krein, Dana Striplin, Nicolle Marinec, Robert D Kerns, Karen B Farris, Satinder Singh, Lawrence An, Alicia A Heapy

**Affiliations:** ^1^ Center for Managing Chronic Disease Department of Health Behavior and Health Education University of Michigan School of Public Health Ann Arbor, MI United States; ^2^ Center for Clinical Management Research VA Ann Arbor Healthcare System Ann Arbor, MI United States; ^3^ Department of Internal Medicine University of Michigan Ann Arbor, MI United States; ^4^ Pain Research, Informatics, Multimorbidities and Education (PRIME) Center VA Connecticut Healthcare System West Haven, CT United States; ^5^ Department of Psychiatry Yale School of Medicine West Haven, CT United States; ^6^ Department of Clinical, Social, and Administrative Sciences College of Pharmacy University of Michigan Ann Arbor, MI United States; ^7^ Department of Electrical Engineering and Computer Science College of Engineering University of Michigan Ann Arbor, MI United States

**Keywords:** Medical Informatics, mhealth, artificial intelligence, comparative effectiveness

## Abstract

**Background:**

Cognitive behavioral therapy (CBT) is one of the most effective treatments for chronic low back pain. However, only half of Department of Veterans Affairs (VA) patients have access to trained CBT therapists, and program expansion is costly. CBT typically consists of 10 weekly hour-long sessions. However, some patients improve after the first few sessions while others need more extensive contact.

**Objective:**

We are applying principles from “reinforcement learning” (a field of artificial intelligence or AI) to develop an evidence-based, personalized CBT pain management service that automatically adapts to each patient’s unique and changing needs (AI-CBT). AI-CBT uses feedback from patients about their progress in pain-related functioning measured daily via pedometer step counts to automatically personalize the intensity and type of patient support. The specific aims of the study are to (1) demonstrate that AI-CBT has pain-related outcomes equivalent to standard telephone CBT, (2) document that AI-CBT achieves these outcomes with more efficient use of clinician resources, and (3) demonstrate the intervention’s impact on proximal outcomes associated with treatment response, including program engagement, pain management skill acquisition, and patients’ likelihood of dropout.

**Methods:**

In total, 320 patients with chronic low back pain will be recruited from 2 VA healthcare systems and randomized to a standard 10 sessions of telephone CBT versus AI-CBT. All patients will begin with weekly hour-long telephone counseling, but for patients in the AI-CBT group, those who demonstrate a significant treatment response will be stepped down through less resource-intensive alternatives including: (1) 15-minute contacts with a therapist, and (2) CBT clinician feedback provided via interactive voice response calls (IVR). The AI engine will learn what works best in terms of patients’ personally tailored treatment plans based on daily feedback via IVR about their pedometer-measured step counts, CBT skill practice, and physical functioning. Outcomes will be measured at 3 and 6 months post recruitment and will include pain-related interference, treatment satisfaction, and treatment dropout. Our primary hypothesis is that AI-CBT will result in pain-related functional outcomes that are at least as good as the standard approach, and that by scaling back the intensity of contact that is not associated with additional gains in pain control, the AI-CBT approach will be significantly less costly in terms of therapy time.

**Results:**

The trial is currently in the start-up phase. Patient enrollment will begin in the fall of 2016 and results of the trial will be available in the winter of 2019.

**Conclusions:**

This study will evaluate an intervention that increases patients’ access to effective CBT pain management services while allowing health systems to maximize program expansion given constrained resources.

## Introduction

### Prevalence and Consequences of Chronic Pain among Veterans

Musculoskeletal disorders are highly prevalent among Department of Veterans Affairs (VA) patients, with chronic back pain the most frequently reported type [1-3]. VA data suggest an annualized increase in the prevalence of low back pain of 4.8% per year due to factors such as an aging population and increasing prevalence of obesity [1,4]. The cost of treating back pain in VA is $2.2 billion annually [2]. Chronic low back pain is associated with work interruption, emotional distress, and risky heath behaviors such as substance use [5]. Emerging evidence suggests that chronic pain compromises successful treatment and management of other chronic conditions [6]. For all of these reasons, increasing access to effective and convenient treatments for chronic low back pain is a national VA priority [7]. Historically, treatment for chronic low back pain has emphasized pharmacotherapy and surgery, while underutilizing evidence-based behavioral approaches that have comparable or superior benefit [8]. Opioid medications are commonly used to manage severe chronic pain, but their use can lead to serious adverse effects [9,10]. Despite its frequent use, there is no evidence of the long-term efficacy of opioid therapy for chronic pain [8].

### Cognitive and Behavioral Interventions to Improve Pain Management

Cognitive behavioral therapy (CBT) is the most widely accepted evidence-based psychological treatment for chronic pain [11,12]. CBT is an attractive alternative to pharmacotherapy because impacts on functioning can last long after treatment is discontinued, and CBT does not entail the negative side effects of opioids. The goal of pain CBT is to assist patients in developing an adaptive problem-solving approach to pain management, and CBT targets both reductions in pain symptoms as well was their associated disability and emotional distress. VA recommendations regarding pain CBT recommend 10 hour-long sessions delivered weekly and focusing on pain education, practice of pain self-management skills, and productive and pleasurable activity and exercise. Skills address both cognitive processes (eg, catastrophizing) and behaviors (eg, relaxation). Meta-analyses have found that CBT has moderate to large effects on pain-related outcomes [13,14].

Because pain CBT is labor intensive and therapists are scarce, many veterans do not have access to these services. A review of data for veterans receiving outpatient opioid prescriptions showed that less than half received any mental health treatment [15], and a survey by VA’s National Program for Pain Management found that half of VA facilities did not have any pain-focused psychological services such as CBT. This suggests that VA needs to identify creative strategies to ensure that patients receive the treatment they need, which could be achieved through a stepped-care model: assigning some patients to interventions with more clinician contact and others to more self-directed interventions.

Ideally, patient feedback could be used to assign patients to the appropriate level of stepped care; however, to date, the use of patient feedback in pain CBT has been suboptimal. For patients receiving pain CBT, retrospective symptom reports are often collected using paper-and-pencil surveys and are vulnerable to recall and social desirability biases; for example, reports may be disproportionately influenced by recent experiences and patients’ emotional states at the time of assessment [16]. Patient feedback is least likely to be available among veterans with the greatest risk for missing in-person sessions, that is, the very patients who may have the greatest need for adjustments in their treatment plan. For all of these reasons, scarce CBT services can be slow to adapt to variation in patients’ treatment response.

Standardization of mental health services such as CBT has improved care relative to unsystematic differences in delivery across patients and therapists; however, new models of CBT need to incorporate a systematic stepped approach to ensure that care is patient-centered, efficient, and targeted to veterans’ unique needs. VA CBT pain treatment is based on evidence that typically reflects average effects in controlled trials, rather than taking into account the substantial variation across patients in treatment response. As such, guidelines are at odds with evidence regarding variability in the characteristics of CBT delivery models with demonstrated efficacy. Recommendations for 10 hour-long CBT pain treatment sessions likely represent the upper bound of what is feasible, given VA budgets and some patients’ limited tolerance for frequent contacts. As many as 25% of patients receiving psychotherapy improve after 1-2 sessions [17], and patients often drop out of treatment that is providing only marginal benefit. Some evidence-based CBT programs have as few as 6 sessions while others have twice that many [18]. Studies from other areas of chronic disease management show wide variation in providers’ recommendations regarding visit frequencies [19,20]. Providers typically make these decisions based on the patient’s perceived stability or expected likelihood of treatment response. However, one study found no correlation between visit frequency and hypertension control [21], and visit intervals can sometimes be substantially lengthened without decreasing quality [22]. No single “dose” of CBT is likely to be appropriate for all veterans, and neither clinicians nor patients may be able to judge *a priori* who needs more resource-intensive forms of care.

### Prior Research on Adapting Treatment to Patients’ Individual Needs

Lambert and colleagues demonstrated the benefits of adapting psychotherapy based on feedback about patients’ progress [23-26]. Other recent work by DeRubeis and colleagues has demonstrated that pretreatment characteristics of patients can be identified that suggest an advantage with respect to the likely response of a given therapy (eg, antidepressant medications versus CBT) and could be used to recommend one course over an alternative [27,28]. While these studies represent an important step toward the goal of tailored treatments, prior efforts to personalize therapy have used patient surveys at the time of intake or (at most) in-person encounters with patients to obtain information about predicted treatment response. As a result, opportunities to adjust therapy have been limited, and the impact of patient tailoring has been modest. Other investigators have suggested that monitoring and feedback could best be accomplished using health IT [29] to allow treatment decisions to be based on real-time information about patients’ functioning. Another key weakness of prior work is that feedback on treatment response is typically provided to clinicians along with nonevidence-based algorithms for modifying patients’ treatment plans [17]. As such, steps toward a more systematic and evidence-based approach to adaptive treatment have been left with a format that cannot respond effectively to real-time information about what works best for each patient.

Another foundational area of research for this study is the theory of tailored health communication, which suggests that patients are more likely to internalize health messages when those messages are relevant to them personally [30]. The state-of-the-science in tailoring uses surveys to identify patients’ needs, health beliefs, learning styles, cultural context, and other factors prior to crafting messages targeting behavioral changes. The data needed to tailor these messages is substantial, and many patients may not be willing or able to accurately report that information at program outset [31,32]. For example, CBT skills training was found to be no more effective when skill presentation was tailored according to what patients thought they wanted before initiating treatment [33]. Also, previous systems typically tailor based on static patient traits, rather than on updated information about patients’ status or treatment response. In this study, we will tailor the intensity and mode of delivering pain CBT services using IVR-reported feedback about patients’ pain-related physical functioning measured objectively via pedometer step counts, perceived functioning scores, and progress with CBT skill practice. Based on this real-time feedback, AI-CBT will personalize each patient’s course of treatment automatically to achieve the greatest benefits for the population, while using clinical resources as efficiently as possible.

### Mobile Health (mHealth) Approaches to Self-Management Support

Because mHealth services have low marginal costs, they can cost-effectively reach large numbers of patients between face-to-face encounters to provide self-management support [34-37]. More than 50 studies have demonstrated that patients can provide reliable and valid information about psychiatric symptoms and substance abuse disorders via IVR and other mobile health technologies [38-41]. The benefits of standard CBT diminish after patients discontinue therapy, and maintenance interventions delivered via IVR sustain those improvements in symptom and self-management skills [37,42]**.** Despite their potential, mHealth interventions typically deliver a simplistic series of messages based on pre-determined “if-then” rules and deterministic protocols. As a result, interactions can feel “robotic” to users and many disengage [43]. In this study, we will test a model to take advantage of the cost and accessibility benefits of mHealth services, while ensuring that these powerful tools are integrated systematically with personal and professional care by trained CBT therapists.

### Conceptual Framework

**Figure 1:**
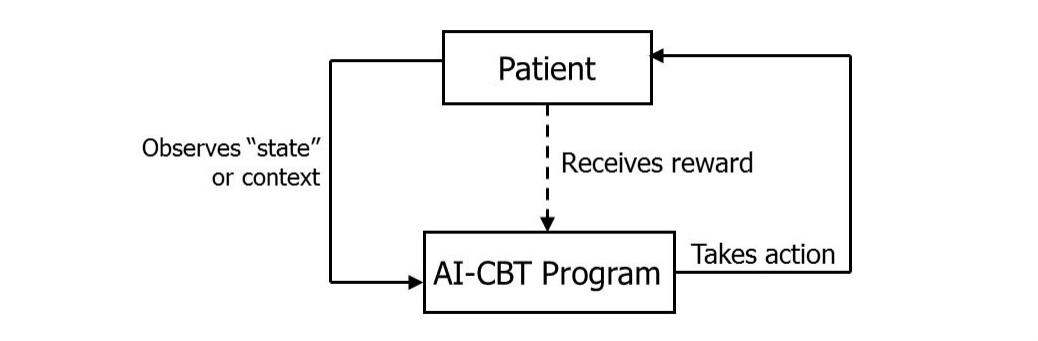
The Reinforcement Learning feedback loop. The AI-CBT actions are the 3 CBT session types; the is IVR-reported pedometer step counts, and state data is IVR-collected information on patients CBT skill practice and pain-related functioning.

The intervention we will evaluate is based clinically on widely adopted and evidence-based models of CBT for pain management (described above) [44], and links those concepts with a strategy for personalized stepped care using *reinforcement learning (RL)*. RL is a field of artificial intelligence that allows an “intelligent agent” to learn what treatment choices work best to optimize a measurable outcome (termed the system’s “reward”; see Figure 1). For readers new to this approach, it is important to emphasize that here we use “learning” to describe the RL system’s progressive statistical adaptation based on patient data, rather than to describe a process through which the patient learns self-care skills by exposure to the intervention. The process used to optimize treatment choices in RL mimics the way that humans learn skills such as riding a bicycle, that is, through systematic adaptation and generalization accompanied by targeted trials of new behaviors with measurable outcomes. RL algorithms similar to those we will apply in this study are the basis of online consumer targeting programs such as Netflix, Google, and Amazon [45], where a service learns automatically how to deliver information that is most relevant to each user. In the current trial, the RL agent will be a computer system that makes weekly recommendations for each patient with respect to the mode and intensity of CBT that the patient should receive (ie, the “actions” that the system can take). Those recommendations will be based on the patient’s progress, progress of similar patients, and other contextual information for that action choice.

Potential actions the AI-CBT program will take include a standard one-hour telephone CBT therapy session, a 15-minute telephone CBT therapy session, and an IVR automated therapy session designed to teach and reinforce skill-based learning. Fifteen minutes was chosen to be consistent with the time increments of the health and behavior CPT codes (15, 30, 45, and 60) used to bill for behavioral interventions for chronic pain. Content for each session type will be based on standard CBT programs for pain management, modified by a panel of experts to be most effective given the length and mode of each contact. The AI-CBT agent’s recommendation regarding which action to take will be based on each patient’s IVR-reported pedometer step counts (ie, the “reward”) as well as other “state” information (Figure 1) also collected via IVR. Importantly, the RL algorithm will learn not only based on each patient’s own treatment response, but will incorporate experience from the response of other patients who have similar characteristics and response trajectories as indicated by the “state space.”

Based on this feedback loop, the RL engine will modify the probability distribution across treatment choices and make recommendations for each patient each week. Because actions will be probabilistic rather than “hard-wired,” the AI-CBT program will avoid potentially overreactive treatment changes that can result when therapists attempt to tailor care nonsystematically or using deterministic flow diagrams. All patients will begin with a standard one-hour CBT session. Based on their progress as measured by feedback on the “reward” and “state” space, patients who progress toward functional goals will be moved through less resource intensive options, and patients who need more intensive follow-up will be moved automatically to more time-intensive, therapist-delivered CBT.

### Prior Work by the Investigators

#### Patient Engagement in IVR Self-Care Support Calls

Dr Piette and his team have more than 15 years of experience developing IVR systems to enhance care for chronically-ill veterans, and over the past 5 years, more than 2000 patients have participated in their IVR programs. In an analysis of data from more than 1200 program participants with 29,000 patient-weeks of follow-up, patients completed 83% of weekly IVR assessment and self-management support calls, and completion rates were similar across groups defined by sociodemographic risk factors [46]. Other recent studies [47,48] found that completion rates of IVR assessments are high among patients with depression, and patients’ IVR reports are at least as reliable as mental health information collected via other methods. Dr Heapy and colleagues also have found high levels of adherence to IVR call schedules in 2 studies among Veterans with chronic pain. In one study, participants with endpoint data completed 85% of expected IVR calls, and participants who withdrew or were disqualified completed 74% of potential calls [49]. In a pilot study designed to obtain feedback from veterans with chronic pain about self-management, 65% indicated a willingness to receive self-management support via IVR, and 11% indicated they might be willing.

#### Impact of IVR Self-Management Support on Outcomes of Chronic Illness Care

In 3 randomized trials directed by Dr Piette, results indicated that IVR call-supported chronic-illness care can improve patients’ self-care and health outcomes [50-52]. In one trial conducted among diabetes patients [51], intervention patients receiving weekly IVR monitoring and self-care support with follow-up by a telephone nurse therapist reported significantly better home glucose monitoring, foot care, medication adherence, and weight monitoring than control patients at their 12-month follow-up. More than twice as many intervention patients had acceptable glycemic control at 12 months (*P*=.01), as well as fewer diabetic symptoms, greater satisfaction with care, fewer symptoms of depression, greater perceived access to care, and greater self-efficacy in managing their self-care (all *P*<.05). In another trial [50], veterans receiving IVR-supported telephone care management reported better self-management behaviors, were more likely to be seen in diabetes-related specialty clinics, had better glycemic control, and reported better patient-centered outcomes.

#### Pedometers for Monitoring Patients’ Physical Activity

Dr Piette was the principal investigator for an NIH-funded randomized trial of telephone CBT plus physical activity promotion among patients with diabetes and depression [53]. Investigators used standard pedometers to objectively measure patients’ physical activity at baseline and 12-month follow-up, and intervention patients also used pedometers as part of their CBT self-management program. We observed high rates of adherence to the collection of pedometer data, and the opportunity to use a pedometer to pursue physical activity goals was an important motivator for trial participation. In another recently completed trial [54], veterans with chronic back pain were randomized to a pedometer-based, Internet-mediated walking intervention or usual care. Intervention participants reported a greater decrease in back pain-related disability in the 6 months following study enrollment. Intervention participants uploaded pedometer data at least once per week for a median of 32 weeks (62% of the recommended time) and more than 25% of participants uploaded data for at least 42 weeks. In summary, we have found consistently that pedometers represent an important alternative to self-reported activity levels, which often have high rates of random reporting error as well as social desirability biases [54-56].

### Preparatory Collaborative Work

We performed simulations to estimate the impact of AI-CBT compared to 10 standard, one-hour CBT sessions delivered by a therapist. We focused on AI-CBT’s impacts on patients’ physical functioning (in this case pedometer-measured step counts) and on therapist time. We assumed that (as in the proposed study) the AI-CBT program would start each patient with a one-hour therapist session and then would automatically develop a personalized step-care program that included additional one-hour “live” telephone therapy sessions, 15-minute live sessions, or IVR sessions. We assumed that patients responding to IVR therapy would also respond to a 15-minute live call or an hour-long call, and patients who responded to a 15-minute therapist call (but not IVR) would also respond to an hour-long session. Simulations evaluated variations in the expected benefit of CBT delivered via different modes, the speed in which patients were recruited into the AI-CBT program (which would affect the system’s ability to learn from prior experience), and whether the AI engine could move a patient directly from a one-hour session to IVR, or whether choices were constrained so that the system would “step down” from one hour to 15 minutes, and from 15 minutes to IVR. We also explored the effect of random error in the expected effect of each CBT session, and the effect of patients’ nonadherence to IVR requests for daily step count data (patients with more missing step count data would be progressed more slowly to less resource-intensive options). See Multimedia Appendix 1 for a summary of those simulations. In brief, using conservative assumptions, we estimate that AI-CBT will be able to achieve an improvement in physical activity that is 93% as great as that seen in standard CBT, but using only 44% of the clinician time required for 10 one-hour sessions for all patients.

## Methods

### Overview

This will be a randomized noninferiority study comparing standard pain CBT to an innovative strategy that uses mobile health technology and artificial intelligence in conjunction with trained CBT therapists to deliver evidence-based, stepped pain therapy so that pain management is as efficient as possible while maintaining the effectiveness of current approaches. Patients in both groups will receive CBT delivered via telephone by the site’s trained pain CBT therapist. For patients in the standard CBT group, the therapist will deliver 10 hour-long CBT sessions based on content used throughout VA. Patients randomized to the AI-CBT treatment group will begin with one standard, hour-long telephone CBT session and will be asked to report their pedometer-measured step counts, pain-related functioning, and CBT skill practice via 5-minute daily IVR calls. Some of those IVR calls also will include reminders regarding the dates and modalities for upcoming CBT sessions. Based on patients’ IVR feedback, the AI-CBT engine will make recommendations to carefully step-down the intensity of each patient’s CBT follow-up using more brief telephone therapy sessions (15 minutes), or IVR therapy. Based on experience gained from each patient’s history and the overall population of patients, the AI-CBT engine will seek to optimize the population’s total improvement in functioning while maintaining each patient at the least resource-intensive mode of CBT delivery. Outcomes will be measured via telephone survey at 3 and 6 months post recruitment, and additional data will be collected via clinical records. We will use data from therapists’ activity logs and administrative files to conduct a budget impact analysis. Additional data to aid translation of study findings from research into practice will be collected via qualitative interviews with CBT therapists, other clinical team members, and patients with various levels of program response.

### Patient Identification and Recruitment

The study will be conducted among patients with chronic low back pain in facilities affiliated with the VA Ann Arbor Healthcare System and the VA Connecticut Healthcare System. Participants who have a diagnosis of low back pain and a pain score of ≥ 4 (indicating moderate pain) on the 0-10 Numerical Rating Scale during at least two separate outpatient encounters in the past year will be identified via electronic medical records. Eligible patients must: (1) report at least moderate pain-related disability as determined by a score of 5+ on the Roland Morris Disability Questionnaire at baseline, (2) report at least moderate musculoskeletal pain for at least 3 of the prior 6 months [57], (3) not be actively psychotic, suicidal, or severely depressed (ie, a score of 20+ on the 9-item Patient Health Questionnaire or PHQ-9 [58]), (4) not report behavior flags related to emotional dysregulation, bipolar disorder, or active substance abuse that could impede participation in the study, (5) be free of life-threatening conditions that could impede participation, such as chronic lung disease requiring oxygen or cancer requiring chemotherapy, (6) be free of dementia defined by a score of 20 or greater on the St. Louis University Mental Status screener [59], (7) have a mobile phone or touch-tone land line phone, (8) be free of sensory deficits that would impair participation in telephone calls, and (9) report that they are not currently receiving CBT and have no plans for surgical treatment related to their back pain. After obtaining agreement from patients’ primary care providers, a letter will be sent to veterans informing them about the study and inviting participation. Veterans who do not opt-out by postage-paid response card will be called by research staff to explain the study, conduct screening, and solicit their involvement. If the veteran is willing, s/he will be sent the consent form by mail along with a postage-paid return envelope. We have used this same process in numerous prior studies and found that it is an efficient and effective way to recruit large samples of veterans without requiring an in-person recruitment visit. The study coordinator will track the percentage of eligible veterans who enroll in the trial and will actively solicit reasons for declining. This information will be used to assess the intervention’s reach, as described in the implementation portion of the application (Aim 3).

In preparation for this study, we used VA Corporate Data Warehouse records for 2012 to identify patients treated in Ann Arbor and West Haven with low back pain (International Classification of Diseases [ICD-9] codes 742.01, 724.02, 724.03, 724.09, 724.1, 724.2, 724.3, 724.4, and 724.5) and a pain score of ≥ 4 on the 0-10 Numerical Rating Scale during at least two separate outpatient encounters. We identified 105,344 patients and estimate that we would have to recruit 3-4% at a rate of 4.4 patients per site per month to reach accrual goals. Our prior studies based on similar populations have recruited 5 to 10 veterans with chronic pain per month per recruiter; therefore, we expect no difficulty recruiting the target sample within the proposed timeframe and staffing.

### Randomization

After completing baseline assessments, patients will be randomized to AI-CBT or standard telephone CBT. Randomization will be done by research staff using sealed opaque envelopes and the computer-generated randomization series. To ensure balance across treatment arms in potential modifiers of the intervention effect, randomization will be done within strata defined by site and age.

### Common Elements of Standard and AI-CBT

#### Overview

Both CBT conditions will involve 10 treatment modules delivered over 10 weeks. The same therapist at each site will provide treatment to patients in both groups. In each arm, the 10-week course of therapy will include an introductory module, followed by 8 pain coping skills training modules and concluding with a final session emphasizing skill consolidation and relapse prevention. The introductory module will present the biopsychosocial model, which explains how chronic pain can lead to dysfunction across numerous domains and provides a rationale for the efficacy of pain coping skills to manage chronic pain. The 8 skills that will be presented were selected based on their efficacy in improving pain outcomes and their appeal to patients in prior trials. These include sessions focused on physical activity, behavioral activation, pacing, and relaxation. We have included modules that address common maladaptive cognitions such as pain catastrophizing and fear of movement or kinesiophobia; a module on sleep hygiene techniques was also included to address sleep complaints that are common among persons with chronic pain and whose treatment has positive effects on pain intensity. Using procedures developed in two previous VA-funded studies, during sessions 2-9, participants will be assigned a goal related to newly presented adaptive pain coping skills (eg, "practice relaxation exercise for 20 minutes daily”) and a daily walking goal (average daily steps over the prior week plus 10%). As participants progress through treatment, they will continue to practice prior goals. In order to maintain equivalence across treatments, participants in both groups will be assigned the same skill practice goals and the same formula will be used for assigning steps goals.

#### Patient and Therapist Materials

Patients in both treatment conditions will use a handbook based on those used in prior trials. The handbook will be identical for both conditions, except that the AI-CBT handbook will contain additional information that describes the three AI modes (one-hour, 15-minute, and IVR sessions) and how to prepare for each type of session. The therapist manuals will be adapted from materials developed for our IVR-based CBT for Chronic Low Back Pain trial. The AI-CBT section will detail specific guidelines for each treatment mode (ie, one-hour, 15-minute, and IVR).

#### Therapist Training and CBT Fidelity

Therapists will be Master’s or doctoral-level clinicians (clinical psychologists or social workers). Therapists will receive 20 hours of training in delivering CBT. Training will include review of the treatment manual, education regarding the nature of chronic pain, the treatment and its rationale, and role-playing the intervention. Therapists will demonstrate mastery of the treatment manual and its procedures by passing a series of quizzes on the module content with at least 85% correct. Therapists will then provide treatment to mock patients and the Co-PI will review the audio-taped sessions, rate fidelity to the treatment manual, and provide feedback to the therapists until they are able to demonstrate proficiency in treatment delivery and adherence to the treatment protocol.

During the intervention trial, all treatment sessions will be digitally audio-recorded. Thirty percent of treatment sessions will be randomly selected and rated for treatment fidelity using the Yale Adherence and Competence Scale (YACS) [60], a validated scale that assesses therapist adherence and competence in delivering manualized behavioral therapy. Because treatment sessions vary in length in the AI treatment condition, we will assess 100% of treatment sessions to ensure that actual session time is within 15% of the AI system-assigned treatment session length (one hour or 15 minutes). Corrective feedback will be given throughout the study to prevent therapist drift.

#### Pedometers for Monitoring Patients’ Physical Activity

All patients will be given a pedometer and a log for monitoring their step counts. We expect to use a Yamax DigiWalker pedometer because it is accurate and used frequently in research [61]. Patients will be mailed a pedometer after completing their baseline assessment and returning their consent form.

### Standard Telephone CBT (Control)

Control patients will receive telephone CBT consisting of 10 weekly modules delivered via one-hour telephone contacts with a therapist. The format of each session will include (1) review of patients’ pedometer logs and coping skill practice, (2) review of previous material and correction of misunderstandings of the information, (3) assignment of new step count goals and discussion of new skills-based material, and (4) discussion of specific step and skill practice goals. Positive feedback and praise will be offered for any skill practice and step goal efforts and accomplishments. Barriers to practice or goal completion will be identified and problem-solving techniques will be used to address them.

### Cognitive Behavioral Therapy Supported by Artificial Intelligence (AI-CBT)

#### Daily IVR Reports

Patients will report their pedometer-measured step counts, CBT skill practice, and pain-related functioning via daily 5-minute IVR calls. Patients will receive calls at times they indicate as convenient and will respond to recorded inquiries using their touch-tone phone. If the initial call is missed, the system will automatically try again 15 minutes later and again 1 hour later. We have successfully used these methods in studies achieving high patient response rates. Pedometer step counts will measure activity over the prior 24 hours, and patients will report their skill practice using a 0-10 scale. Pain-related functioning will be assessed using a single item from the West Haven-Yale Multidimensional Pain Inventory (WHYMPI) [62].

Step counts will be used in the “reward function” that the RL algorithm will seek to optimize, and skill practice and physical functioning reports will be used as “state” information that the system will take into account when making decisions that optimize patients’ treatment course. The definition of the reward function is of course a crucial decision in AI, since all action choices will be evaluated in terms of whether or not they optimize that goal. We chose step counts because they represent an objective measure of patients’ physical function, a direct behavioral target of CBT, and an outcome of pain CBT programs as defined by national guidelines. Perceived pain-related interference, sleep quality, and other subjective experiences of chronic pain syndromes also will be collected via IVR and periodic surveys. In post-hoc analyses, we will be able to evaluate whether a weighted composite reward (eg, taking both steps and symptoms into account) might lead to more efficient optimization and more effective action choices. The AI system will be able to accommodate missing IVR reports, and patients who fail to complete more than 50% of the daily IVR calls in a 2-week period will be called by a research associate to troubleshoot problems and encourage compliance with feedback. In addition to being the source of data with which AI-CBT will personalize each patient’s course of treatment, data from IVR calls will be used to inform therapists of participants’ treatment adherence and progress. These data will be particularly important for informing abbreviated 15-minute therapist sessions when priority is placed on the efficient use of treatment time and during IVR sessions when the entire session is pre-recorded. Once a week, the IVR call will include a brief weekly message alerting patients of the date, time, and modality for their subsequent week’s session.

#### AI-CBT Action Recommendations

After Week 1, session options will include (a) one-hour telephone treatment sessions, (b) 15-minute live telephone therapist sessions, and (c) IVR treatment sessions. To avoid scheduling conflicts, AI-CBT patients will be assigned a one-hour block of time each week in which both they and the CBT therapist are available for treatment. This same time slot will be used for either the hour-long therapist sessions, the 15-minute therapist sessions, or the IVR CBT sessions. Each Monday morning, the CBT therapists will receive a list of AI-CBT personalized treatment recommendations for that week for each patient. By noon on Monday, the therapist will have a finalized schedule of which patients require what types of contact that week, and which patients need to have a summary of the therapist’s comments and recommendations recorded for the week’s IVR CBT therapy call.

During *Week 1*, all patients in AI-CBT will have an hour-long telephone session with the CBT therapist. During that session, the therapist will review the goals and process of the program and will present the standard introductory material contained in Session 1 of the standard CBT program. The *one-hour AI-CBT sessions* will be identical to those of the control condition (see above for details) and will follow the same progression of content used for control patients. The *15-minute telephone CBT sessions* will mirror the content of the one-hour sessions, though in a compressed form. Protocols in the therapist manual and patient handbook will emphasize the importance of using session time efficiently and using a consistent format that includes reviewing the patient’s daily IVR reports, clarifying patient handbook information regarding the current week’s adaptive pain coping skill, and setting goals for skill practice and step counts for the coming week. Prior to the session, therapists will review patient-reported information collected via daily IVR calls. If participants have not been successful in meeting step or skill practice goals, the therapist will help the participant identify barriers to goal attainment and use problem-solving strategies to address barriers. The therapist then will ask the patient to describe the current week’s adaptive pain coping skill as a brief check of their understanding and will clarify any misunderstood information. Finally, the therapist will review goals for the coming week and discuss any anticipated barriers to meeting goals. Any remaining time will be used to review the skill and to encourage the patient to read their patient handbook. Much of the content for the *IVR CBT sessions* has been developed and implemented as part of our ongoing IVR-based CBT for Chronic Low Back Pain trial. Our experience in that trial suggests that patients complete the IVR sessions more than 90% of the time and that satisfaction rates are high. During these sessions, patient will receive 2-5 minutes of pre-recorded feedback from their therapist, during which the therapist will review the patient’s recent IVR-reported changes in step counts, pain-related functioning, and skill practice. Reinforcement will be provided for effort, and improvements will be noted. IVR messages will include a review of the pain coping skill practice and step goals for the coming week, and participants will have the option of leaving a message for their therapist via the IVR system, should they have a question. Therapists can leave a response message, also on the IVR system. These IVR CBT sessions typically take 15 minutes to complete.

#### The AI Engine

Patients’ IVR-reported step counts, skill practice, and pain-related functioning will be accessed by the AI engine daily to update the probabilities that the system uses to determine which treatment step to recommend for each patient the next week. We will use a state-of-the-art AI algorithm (LinUCB) designed to make careful choices while learning quickly from a patient’s treatment response as well as the experience of other patients with similar characteristics [45]. With increased interactions, the system will learn to tailor these decisions more effectively to maximize population-level improvements in functioning while minimizing clinician time. In this way, AI-CBT will function similar to the best clinicians, who learn from experience within and across patients to improve their care. In the context of the trial, this means that patients enrolled early will likely receive less personalized CBT courses that are relatively similar to the standard CBT approach (ie, with a greater number of hour-long sessions), while patients enrolled later will receive services that are more personalized and include a greater frequency of 15-minute therapist sessions and IVR sessions. To maximize the efficiency of this “learning curve,” (1) patients will be recruited over a longer period than would potentially be necessary, so that the AI-CBT program can gain as much experience as possible from patients recruited first and apply that knowledge to patients entering the program later, and (2) patients will be randomized with a greater “N” in the AI-CBT group so as to maximize the system’s experience (see power calculation, below). As part of our evaluation, we have planned an a priori subgroup analysis in which we will compare randomization groups on each specific aim separately for early versus later enrollees, to test the hypothesis that AI-CBT will result in greater benefits over time. These analyses also will allow us to estimate program benefits if AI-CBT were implemented with thousands of patients and multiple years of experience.

#### CBT Treatment Fidelity

CBT treatment fidelity will be assessed using a modified version of the Yale Adherence and Competence Scale [60], a validated scale that assesses therapist adherence and competence in delivering manualized behavioral therapy. Dr Heapy will rate audiotapes of 30% of all CBT therapist sessions to assure that treatment is consistent with the manual and will provide corrective feedback to therapists whenever drift occurs.

#### Role of the Expert Panel

The AI-CBT program will be supervised with ongoing input from an expert panel comprised of experts in pain management, CBT for chronic pain, clinical trials using behavioral interventions, adaptation of therapy materials for telephone delivery, and IVR. The panel will meet several times by teleconference during the study start-up period to review and revise the proposed treatment materials and refine the AI algorithm to reflect any constraints that should be put into place to limit the choices that the RL algorithm can make, for example, “if the patient’s physical activity level decreases more than 20% during 2 weeks in a row, recommend 2 hour-long CBT sessions regardless of what their prior week’s contact was.” Experts will meet by teleconference quarterly and in ad hoc sessions if important concerns or questions arise during the intervention.

### Measurement

#### Overview

We have selected outcome measures based on recommendations from the Initiative on Methods, Measurement, and Pain Assessment in Clinical Trials (IMMPACT) [57,63]. Endpoint measures are consistent with CONSORT guidelines recommending that equivalence trials use outcomes that are similar to those used in efficacy studies. We also will examine treatment satisfaction, treatment credibility, patient engagement and dropout, and goal accomplishment. Process and outcome data will be collected via the following sources: (1) *Patient surveys* will be conducted at baseline, 3 months, and 6 months via telephone by trained research assistants. Participants will receive a $20 incentive for each interview completed; (2) *Qualitative interviews* will be conducted with purposive samples of patients in the AI-CBT group at follow-up. We will target patients who demonstrate significant improvement, patients who were very satisfied with AI-CBT, patients without significant improvement, patients who were dissatisfied, and patients who dropped out of the intervention. Additional qualitative interviews will be conducted at follow-up with CBT therapists and clinician team members; (3) *CBT therapist logs* will be used to track therapist time spent in patient treatment and in attempting to reach patients, as well as key information about the content of those interactions; (4) The *AI-CBT IVR system* will automatically capture information about intervention patients’ pedometer-measured step counts, pain-related functioning, CBT skill practice, and missed data reporting events; and (5) *Administrative and clinical data systems* will be used to track patients’ use of other VA inpatient and outpatient services for pain management, mental health, and medical care.

#### Primary Outcome

The 24-item Roland Morris Disability Questionnaire (RMDQ) is an IMMPACT endorsed measure [63] of pain-related disability for persons with chronic low back pain. Strong evidence supports the RMDQ’s reliability, validity, and responsiveness to change during trials [64].

#### Secondary Outcomes

*Global pain intensity* will be assessed using the Numeric Rating Scale (NRS-I) an IMMPACT-recommended 11-point numeric rating scale of pain severity [57]. *Pain-related interference* will be measured using the 9-item Interference subscale of the West Haven-Yale Multidimensional Pain Inventory (WHYMPI). This IMMPACT-recommended measure assesses pain-related interference in daily activities and has demonstrated good internal consistency [57,62]. *Emotional functioning* will be assessed using the 65-item Profile of Mood States (POMS) [65], which is designed to assess six dimensions of mood. Internal consistency and test-retest reliability for the POMS are good, and it requires only 3-5 minutes to complete. *Depression symptom severity* will be assessed using the 21-item Beck Depression Inventory (BDI), a widely used measure with excellent internal consistency and stability [66]. The BDI takes 5-10 minutes to complete. The Patient Global Perception of Change scale is a single-item measure that quantifies a participant’s overall *perception of improvement* since beginning treatment and the clinical importance of that improvement. Participants indicate improvement on a 7-point "much worse" to "much better" scale. This is a well-validated measure recommended by IMMPACT [63]. Finally, we will use the Veterans SF-12 to assess *health-related quality of life.* This measure has demonstrated good internal consistency and is strongly correlated with socioeconomic status and morbidities [67].

#### Resource Use (Aim 2)

To assess *intervention costs*, therapists will use a log to record time spent in intervention-related activities, including patient treatment and consulting with other care providers, for a random 20% of all days for which they are treating patients. These time records will be combined with wage data from the VA Financial Management System to estimate intervention-specific personnel costs. Technology costs of the AI-CBT program include fixed costs (eg, software development and computer maintenance) plus variable costs (eg, minute costs for IVR calls). One-time fixed start-up costs will be reported separately. *VA inpatient and outpatient service use* data will be obtained from the Musculoskeletal Diagnoses Cohort (MSD). The MSD is developing validated algorithms for using VA electronic health record data to identify utilization events, comorbid conditions, receipt of opioid medications, and pain screening results, for patients with pain-related diagnoses. The primary data source is the National VA Corporate Data Warehouse (CDW), which contains electronic pharmacy data, and inpatient and outpatient encounters. The MSD soon will include data from sources currently transitioning to the CDW, such as Decision Support System National Data Extracts (which include the Outpatient and Inpatient Encounter files). Information on non-VA admissions will be collected by the patient survey. To mitigate recall bias, we will use a 2-timeframe method that asks about utilization over the past 6 months and past 2 months, with more weight given to the shorter timeframe [68].

#### Treatment Satisfaction and Engagement (Aim 3)

For patients in the AI-CBT group, we will calculate *IVR adherence* as we have in the past [46], that is, as the proportion of days during which an assessment was attempted in which one was successfully completed and the number of weeks during which the patient completed at least 4 out of 7 requested IVR reports. Participants’ judgments of *treatment credibility* will be assessed using a reliable questionnaire adapted from Borkovec and Nau [69]. Treatment credibility has been shown to be significantly associated with treatment satisfaction, engagement in treatment, and number of sessions attended. The Pain Treatment Satisfaction Scale of the Patient Outcomes Questionnaire will be used to assess *patient satisfaction* with various domains of pain care [70]. This 5-item measure shows good internal consistency and significant associations with staff and patient ratings of patient improvement. To understand *attendance in “live” telephone CBT sessions and program dropout*, we will attempt to reach samples of patients with low levels of engagement for qualitative interviews. Participants will rate their *continued skill use* at follow-up for each of the target behaviors emphasized in the CBT program on a 0 (not at all accomplished) to 10 (completely accomplished) scale. These survey items will be based on those we have used successfully to collect similar data from veterans during IVR assessment calls. As described above, *daily IVR calls* will be used to collect data in the AI-CBT condition regarding pedometer measured step counts, CBT skill practice, and pain-related functioning using pre-recorded questions we have used successfully in our prior studies.

#### Demographics and Covariates Measured at Baseline

Demographics and other covariates have been selected to be consistent with data collected in prior trials of chronic pain and pain-related CBT, including factors that can influence important mediation and moderation processes such as access to care, barriers to enacting behavior changes, and substance abuse and mental health comorbidity. Variables will reflect characteristics predicting psychological treatment response in the Personalized Advantage Index, that is, marital status, employment status, life events, comorbid personality disorder, and prior experience with medications [28]. We will measure patients’ baseline *sociodemographic and pain characteristics* that have been shown to be associated with treatment outcomes such as age, sex, education level, racial/ethnic background, marital status, occupational status, pain duration, and number and location of pain sites. We also will gather data on participants’ level of health literacy [71]. *Psychiatric and substance abuse comorbidities* will be measured using medical record diagnoses and mental health encounters. Additional self-report information will be collected using subscales of the Mini International Neuropsychiatric Interview (MINI) [72] related to mood and substance abuse disorders. *Pain medication use* will be assessed through patient surveys and a review of computerized pharmacy records. Pain medication will be coded as non-steroidal anti-inflammatory, non-narcotic analgesics, narcotic analgesics, and benzodiazepines and other sedative/hypnotics. For each category, ratings also will be made post-treatment, to determine whether patients have experienced an increase, no change, or decrease in their medication use. *Distance from VA* will be calculated using Google maps and used as a measure of geographic access. The *Pain Catastrophizing Scale* is a 13-item self-report scale that examines thoughts and feelings people may experience when they are in pain including rumination, magnification, and helplessness [73]. Finally, *pain-related fear* will be measured using the Tampa Scale of Kinesiophobia-revised (TSK-R), which has two subscales (Fear of Harm/Activity Avoidance and Pathophysiological Beliefs) and has been shown to be sensitive to treatment-related change.

### Sample Size and Power Calculation

To ensure that the AI-CBT program retains a clinically relevant effect relative to standard CBT, the noninferiority margin was set at a 2-point reduction in pain-related disability as measured by the Roland Morris Disability Questionnaire (RMDQ) [64]. A 2-point reduction in the RMDQ is considered to be a minimally clinically significant effect [74]. To detect noninferiority within a margin of 2 points (SD 4.5) with 90% power and Type I error (1-sided) of .025, we will need 108 participants in each group after attrition. If we assume 20% attrition, we would need to randomize 135 patients to each study arm, for a total of 270 patients enrolled. However, given that we expect no difficulty recruiting sufficient numbers of patients, our target sample size will be 320. The additional 40 patients, that is, (320 - 270) - 10 dropouts, will allow us to ensure that we are well powered to detect inferiority in the event of a higher dropout rate, and also will allow us to randomize patients to the AI-CBT versus standard CBT groups using a ratio of 1.37:1. The additional patients in the AI-CBT group will have the added benefit of allowing the AI engine to improve its ability to personalize patients’ stepped care program as quickly as possible.

### Analysis

#### Baseline Comparability

We will examine baseline differences across groups in measures of study endpoints as well as other potential prognostic indicators, such as patients’ age, comorbid diagnoses, and history of pain treatment. Any differences across groups in baseline characteristics will be controlled statistically in analyses comparing outcomes.

#### Intervention Reach and Sample Representativeness

The RE-AIM framework (ie, Reach, Efficacy, Adoption, Implementation, and Maintenance) is a methodology for systematically considering all strengths and weaknesses of an intervention to better guide program planning [75]. To evaluate reach, we will ask patients who decline study participation whether they would be willing to provide informed consent to participate in a brief survey that identifies their reasons for declining participation and the characteristics that differentiate them from enrollees.

#### Analysis of Endpoints (Addressing Specific Aim 1)

We will compare standard telephone CBT to AI-CBT on the RMDQ pain intensity scores at the 12-week follow-up using a one-sided, 2-sample t-test. Because intent-to-treat analysis can raise the risk of Type I error in a noninferiority trial [76], we will conduct both a per protocol and intent-to-treat analysis. Analyses of all other outcomes will be conducted on an intent-to-treat basis. Because the more efficient AI-CBT program will be less burdensome to patients, engagement and outcomes could actually be superior to standard CBT. Our primary outcome analyses will be able to detect superiority in the AI-CBT group, although the study has been designed to detect noninferiority. We expect that RMDQ scores will be normally distributed. If not, we will use transformations to achieve normality. We also will develop a 2-level mixed linear model that uses both RMDQ follow-up scores as the dependent variables; treatment group, time, and the treatment by time interaction as categorical explanatory variables; and baseline RMDQ score as a continuous covariate. This model will also allow for adjustment for design-related factors (eg, site and age). Age will be examined as a blocking variable within 5-year age groups, because randomization will be done within site x age block strata. An unstructured variance-covariance matrix will be used to model the error variance. Secondary outcomes including pain intensity, emotional functioning, global perception of change, and quality of life will be analyzed in a manner similar to that used for the primary outcome.

#### Intensity of Service Use (Specific Aim 2)

We will compare service utilization by category (eg, CBT therapist time, PCP visits, and pharmacy use) between groups. We will conduct a budget impact analysis [77] from the perspective of the VA medical center and will include the cost of the intervention (personnel, supplies, CBT therapist training, and IVR fixed/variable costs) as well as costs for specific medical care services likely to be affected by the intervention. Data from CBT therapists time records will be combined with wage data from the VA Financial Management System to produce estimates of intervention-specific personnel costs. Costs associated with the use of specific medical care services, such as medications, will be obtained from the Decision Support System (DSS) files. Cost analysis will be conducted in accordance with the guidance provided by Mauskopf et al [77], including the use of sensitivity analysis and scenarios that allow for varying assumptions about intervention uptake, compliance, or component costs. All resource use and cost comparisons will be adjusted for any observed baseline differences in patient characteristics. Because costs of resource utilization are usually skewed, alternative modeling techniques (eg, log-transformed costs, negative binomial regression) will be used.

#### Intervention Engagement and Satisfaction with Care (Specific Aim 3)

As in our prior research [46], we will conduct extensive analyses of the process of intervention delivery in both arms. We will monitor the proportion of telephone CBT sessions that are completed, and determine the patient and session characteristics associated with patients’ reports of skill practice. Patients in the AI-CBT group will report their satisfaction with aspects of the intervention (eg, whether it provided information useful for achieving behavioral targets), and satisfaction ratings will be correlated with measures of intervention engagement, patients’ baseline characteristics, and changes in pain-related functioning. Differential dropout across experimental conditions will be examined using Kaplan-Meier curves and survival models.

#### Preplanned Subgroup Analysis

Because AI-CBT will continue to learn patterns in patients’ experience throughout the intervention period, we hypothesize that the second 50% of patients randomized will show an even larger difference in clinician time than the first 50%, while still maintaining near equivalence in pain-related outcomes. Differences in pain-related functioning and in clinician treatment time across treatment groups will be tested in this subgroup analysis after stratifying the sample into early versus later recruits.

#### Approach to Missing Data

If more than 15% of a covariate is missing, we will use multiple imputation methods based on the SAS MI Procedure [78]. Specifically, we will model patients' likelihood of having data and define strata within which values are missing at random. We will then stratify patients according to these propensities, randomly sample from the observed outcome distributions, and impute these values for missing data within each stratum. When data are missing for items within scale scores, we will use recommended imputation procedures rather than deleting patients list-wise from the analysis.

#### Mediators and Moderators of Intervention Effects

We will use multivariate modeling to identify the mechanisms through which the intervention achieves effects on outcomes and whether there are differential effects across subgroups [79]. Initial models will include only treatment group as the predictor. Subsequent nested models will introduce potential mediators (such as the amount of time spent via telephone with CBT therapists), and we will evaluate changes in the relationship between experimental condition and outcomes before and after covariates are introduced. Analyses of effect moderation will focus on baseline pain severity and comorbid diagnoses using standard approaches to evaluate interactions between these covariates and patients’ experimental condition [80]. Significant interactions will be interpreted by plotting regression lines for predicted outcomes of patients with high and low values of the moderator.

#### Evaluating the Reliability of Patients’ IVR Reports

We will evaluate the integrity of IVR-reported step counts and functioning by examining associations between IVR reports and patients’ baseline characteristics that the literature suggests would be associated with patients’ functioning (eg, baseline SF-12 scores, comorbid medical diagnoses, and age). We also will examine serial correlations across IVR reports under the assumption that all correlations between scores and proximal scores should be positive and roughly of equal magnitude, controlling for the time difference between reports.

#### Evaluating Intervention Adoption, Implementation, and Maintenance

These dimensions of an RE-AIM evaluation [75] will be assessed as follows. Adoption will be evaluated by examining variation in study participation and intervention engagement across sociodemographic and clinical subgroups of eligible patients. For example, we will determine whether older patients or those with less education have more difficulty responding to queries about their step counts or other aspects of pain-related functioning via IVR. Adoption at the provider level will be monitored by recording the proportion of primary care providers who are willing to have their patients participate in the trial and providers’ reasons for not participating. Implementation and maintenance will be evaluated through semi-structured questions at follow-up designed to identify program characteristics that might be a barrier to patients’ use of the intervention in other settings and intervention characteristics that patients feel would make it more valuable to others with chronic pain. We also will meet with clinicians at each site to gauge their willingness to adopt and maintain a similar intervention, and the ways such a system can be designed to best complement existing services.

#### Qualitative Interviews and Mixed-Methods Analysis

We will use audio-taped interviews with 20 patients (15 from the AI-CBT arm), CBT therapists (N=2), and clinician team members (5 from each of the two recruitment sites) to provide a context for interpreting intervention effects and suggest additional subgroup analyses. The focus of patient interviews will be on satisfaction with pain care, barriers and facilitators of pain management, and motivation for making behavior changes using automated systems. AI-CBT patient interviews will focus on patients’ satisfaction with the adaptive intervention and the extent to which patients felt that it was able to provide them with the care they needed while using their time effectively. Staff and clinician interviews will focus on barriers to recruitment and maintenance in a larger-scale roll-out of the intervention and the extent to which staff feel that services like this are feasible and useful given their workflow. Staff will be interviewed by the two study PI’s, and patients will be surveyed by a research associate with training by an expert in qualitative analysis. Interviews will be transcribed verbatim, and 20% of the transcripts will be verified by comparing the transcript to the audiotape. We will enter the transcripts into NVivo, for file storage and selective retrieval. Using accepted techniques [81], two reviewers will independently read transcripts, approaching the data with analytic categories in mind, but also identifying other categories in the data. An iterative process will be used until agreement is reached on categories and their definitions, after which we will develop a coding template and enter it into NVivo as a tree diagram. Dr Heapy has experience in gathering and analyzing qualitative data on CBT goal setting and outcomes, and the University of Michigan Center for Managing Chronic Disease has extensive expertise in this important focus of implementation science.

## Results

The trial is currently in the start-up phase. Patient enrollment will begin in the fall of 2016 and results of the trial will be available in the winter of 2019. If successful, the study will establish a new approach for using artificial intelligence to improve pain care. Similar methods could be used to improve the efficiency of chronic disease management services for patients with depression, hypertension, diabetes, and other priority conditions.

## Discussion

### Expected Findings

The AI engine will only make productive decisions about patients’ subsequent therapy sessions to the extent that it has valid, reliable, and current information about patients’ progress. In this respect, the AI-CBT program is identical to clinicians who must rely on patients’ feedback about behaviors such as adherence, to judge treatment response after a change in management. We will examine patients’ IVR reports carefully as described above to identify aberrant patterns that need to be taken into account when evaluating the intervention’s effectiveness and potential of this system for broader dissemination. The AI engine will also be programmed to disregard reports of dramatic changes in patients’ step counts that are likely to be inaccurate. These reports will be treated as missing data, thereby making conservative decisions that leave patients in the relatively intensive treatment modes. Missing data on step counts and CBT skill practice will result in conservative choices in the AI-CBT group, which in the extreme will leave patients with weekly one-hour telephone CBT identical to that received by patients in the control arm. In contrast to other applications of reinforcement learning, for which AI systems can receive millions of “reward” indicators across users in short intervals of time (eg, purchase decisions among Amazon users), the AI engine in this intervention will only be receiving data on a relatively small number of patients and time points. As such, the system will learn relatively slowly for a given patient, especially for patients enrolled in the initial phases of recruitment. We expect that this will lessen the system’s ability to maximize cost-savings by offering less resource-intensive but equally effective alternatives to extended telephone CBT sessions. As such, the differences in per-patient treatment cost across groups will be a lower bound of what could be expected if the service were implemented with larger samples of patients over longer periods of time.

### Dissemination and Implementation Plan

We will engage clinicians at both sites during regularly scheduled primary care meetings to discuss how to make the service most impactful and consistent with their workflow. We also will disseminate short newsletter-style emails with information about the study, and will make PowerPoint presentations and videos of educational presentations available via the National Pain Program Office website. Of particular relevance to engaging other pain researchers is the Pain Research, Informatics, Medical comorbidities, and Education (PRIME) Center, directed by Dr Robert Kerns. PRIME Center resources will be made available to assist with this dissemination plan. An additional avenue for dissemination is the National Pain Research Working Group, which includes pain investigators that teleconference regularly to identify priorities for pain research and develop collaborative projects. The PRIME Center has a well-established collaboration with the Health Services Research and Development (HSR&D) Center for Information Dissemination and Education Resources (CIDER) and the Employee Education System. These will be leveraged to further target policy makers through cyber seminars and other dissemination strategies. We believe that AI-CBT will be an exciting alternative for consumers because it will be less burdensome than standard care and will automatically personalize each patient’s treatment course.

## Appendix

Multimedia Appendix 1 Reinforcement Learning: description of the adaptive algorithm with simulations.

Multimedia Appendix 2 Funding confirmation from the VA Health Services Research and Development Program.

## Abbreviations

AI: artificial intelligence
BDI: Beck Depression Inventory
CBT: cognitive behavioral therapy
IVR: interactive voice response
mHealth: mobile health
PCP: primary care provider
POMS: Profile of Mood States
RL: reinforcement learning
VA: Department of Veterans Affairs
WHYMPI: West Haven Multidimensional Pain Inventory
