# Supplementary material for: Patient-Centered Pain Care Using Artificial Intelligence and Mobile Health Tools: Protocol for a Randomized Study Funded by the US Department of Veterans Affairs Health Services Research and Development Program
Source: JMIR Res Protoc. 2016 Apr 7;5(2):e53. doi: 10.2196/resprot.4995 (PMC4856067; doi:10.2196/resprot.4995)
Supplement: Multimedia Appendix 1 [file resprot_v5i2e53_app1.pdf]

Appendix A:

AI Program: Simulation Methodology

Background

The purpose of these simulations was to estimate the potential impact of AI-CBT relative to 10 sessions of standard CBT, with respect to improvements in patients' pedometer-measured step counts and the amount of CBT counselor time consumed. The simulations presented below were part of a much larger set of simulations we explored, varying factors such as: the expected distribution of patients in the trial (e.g., how many would be responders to IVR versus 10-minute versus hour long CBT sessions), the expected impact of each week's session on patients' step counts, the relative value of counselor time saved, and the mode choices from which the AI-engine was allowed to choose each week.

Assumptions

Here, we assumed that the patient's daily step count after receiving a mode of treatment in a given week could be characterized by the following equation:

$$\begin{aligned} & daily\ step\ count \\ & \quad = last\ week's\ avg\ daily\ step\ count \\ & \quad + \mathrm{p}(patient\ responds\ to\ mode\ x \mid patient\ receives\ mode\ x) * boost + noise \end{aligned}$$

Where the mode effect, "beta", was assumed to be an 8% increase in the patient's daily step count, while the noise parameter represents random variation around that mean increase. We further assumed that each patient's baseline step count is drawn from a Gaussian distribution with mean of 5000 steps per day with standard deviation of 200 steps. The results below are shown for three different assumptions regarding the noise:

(i) Noise free, i.e., that the Veteran always responds to a mode that he is a responder to.

(ii) Small noise, where the noise is drawn from a Gaussian distribution centered at 0 with mean 50. Also, we assume that the patient responds with probability 0.95 given that he is a responder to that mode.

(iii) Larger noise, where the noise is drawn from a Gaussian distribution centered at 0 with mean 200. Also we assume that the Veteran responds with probability 0.9 given that he is a responder to that mode.

For each simulation presented here, we assumed that the population is comprised of equal proportions of patients who respond to the following delivery modes:

1) One hour clinician CBT call
2) 10 minute clinician CBT booster call
3) IVR call
4) No call (to capture temporal trends and longer-term beta effects)

We further assume that patients who respond to a given delivery mode also will respond with equal beta to all other delivery modes above that one in the hierarchy.

The reward received by the AI-CBT agent was calculated by subtracting the total cost of the clinician time used (where the cost per minute is 5 steps) from the average daily step count reported that week. Combining steps and clinician time into a single metric is important so that the AI engine can differentiate the benefits of a fixed improvement in steps achieved via an hour long session versus 10-minute booster (for example).

The average increase in step counts over the ten week course of CBT is reported as a percentage increase in step counts over baseline scores, i.e., (10 week steps-baseline steps)/baseline steps) as a function of the expected increase if all patients received 10 weeks of standard 1-hour CBT sessions.

We used the LinUCB reinforcement learning algorithm. We used for "state" or contextual information whether the patient's response during the prior two exposures to each delivery mode was either "good" (step count increase of 5% or more), "fair," (increase >0% but less than 5%) or "poor," (step count change of $\leq$ 0%).

We examined two alternative constraints on the choices available each week to the AI engine:

For Action Choice #1 (a1 in the figures below), the AI engine was allowed to select each week from all three action choices, i.e., 1 hour counselor session, 10 minute counselor session, or IVR, plus a no-call option each week.

For Action Choice #2 (a2 in the figures below), each patient was started with a one-hour counselor session, and then after that, the AI engine was only allowed to choose to either stay at the current level the subsequent week, or step-up and step-down one level in the hierarchy base on the patient's response in prior weeks. Also for a2, we allowed the AI engine to consider as additional state information whether the patient had ever stepped back up to a more time-intensive mode as the result of unsatisfactory improvement in step-counts. For patients with one prior failed step-down, the AI engine was programmed to maintain the patient at the same level regardless of the patient's subsequent response.

Procedure

The simulations for Action Choices #1 and #2 were conducted similarly: First a probability distribution determining the mode choice for each patient in each week (i.e., the "policy" in reinforcement learning terms) was learned using populations of (n=20, n=50, n=100, and n=350). We assumed that patients were recruited for the n=350 sample in batches of 50 patients to maximize learning. Next, the policy was evaluated based on a new population of 100 patients (with the same distribution of impact expectations as the training sample) for 10 weeks. A higher value for alpha means that the agent does more exploration. The graphs below show the results averaged over 100 runs, where one run represents one patient-week. The best performing parameter values for each of the mode choices were compared against the policy of always making either a one hour call, a 10 minute call, an IVR call or making no call at all (i.e., the various mode choices that would be tested if this were a standard randomized trial in which all patients were forced to receive care using a fixed mode type).

Results Assuming No Noise in Beta

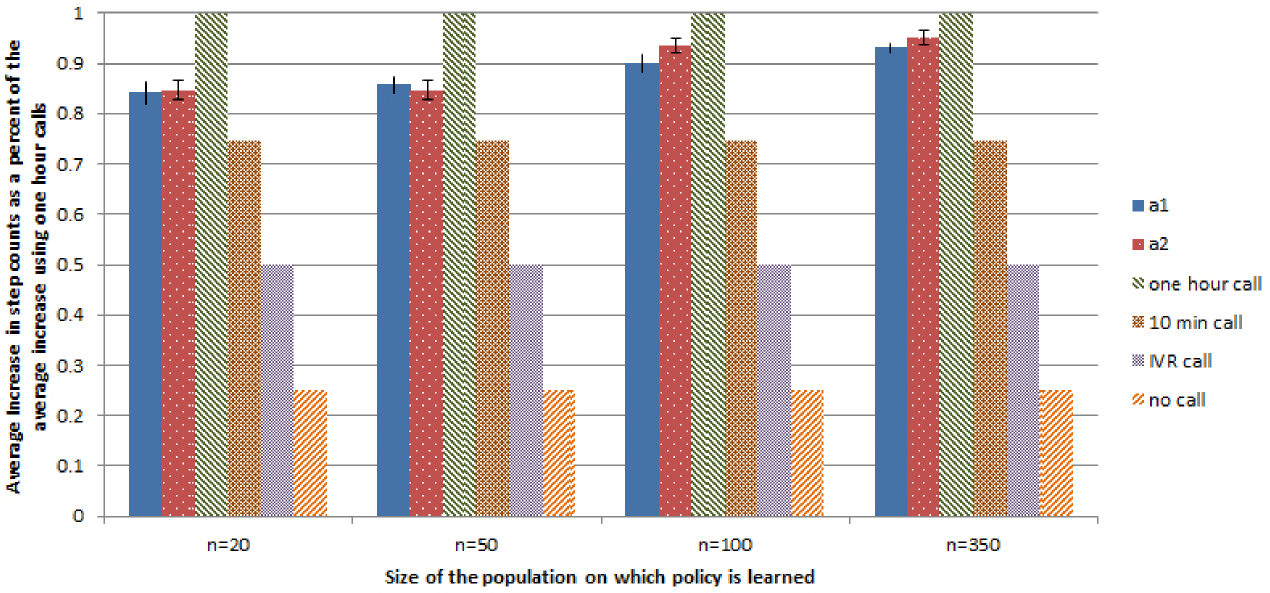

Figure 1: Average Increase in Step Counts as a Percent of the Expected Average Increase Using One Hour Calls (assuming no noise)

We see that the system increases performance as the size of the population in the learning phase is increased. For the policy learned using a population of 100 patients, the AI engine using Action Choice #2 (i.e., a2) achieve approximately 93% of the improvement in step-counts compared to that expected if all patients received only one-hour calls. For a policy learned using a population of 350 patients, that relative improvement increases further, although not substantially (to 95%). It is important to note that because the AI engine is only using the performance of that patient as “state” information in deciding which action to take, it will have to make some mistakes in order to learn what does not work for each patient and therefore cannot achieve 100% of the improvement that would be expected if all patients were given one hour sessions regardless of the other modes to which they would respond. Including more information in the “state” that would allow the AI engine to generalize experience from one Veteran to another would improve performance even more. It is also noteworthy that Action Choice #2, in which the agent was constrained to make more conservative moves across modes, achieved better average weekly step count increases than Action Choice #1.

Figure 2 shows that the increased expected effectiveness of Action Choice #2 comes with a cost, since most patients on average will be expected to consume more clinician time with an approach in which they are gradually stepped-down. ***However, focusing concurrently on the results shown in Figures 1 and 2 – and concentrating on the bars for “a2” (i.e., Action Choice #2), the simulations suggest that even with more gradual step choices across modes, Action Choice #2 will achieve between 90% and 95% of the benefit in terms of increased step-counts, with ~40% to 45% of the clinician time that would be used if all patients were automatically assigned to a full course of 10 one hour counseling sessions.***

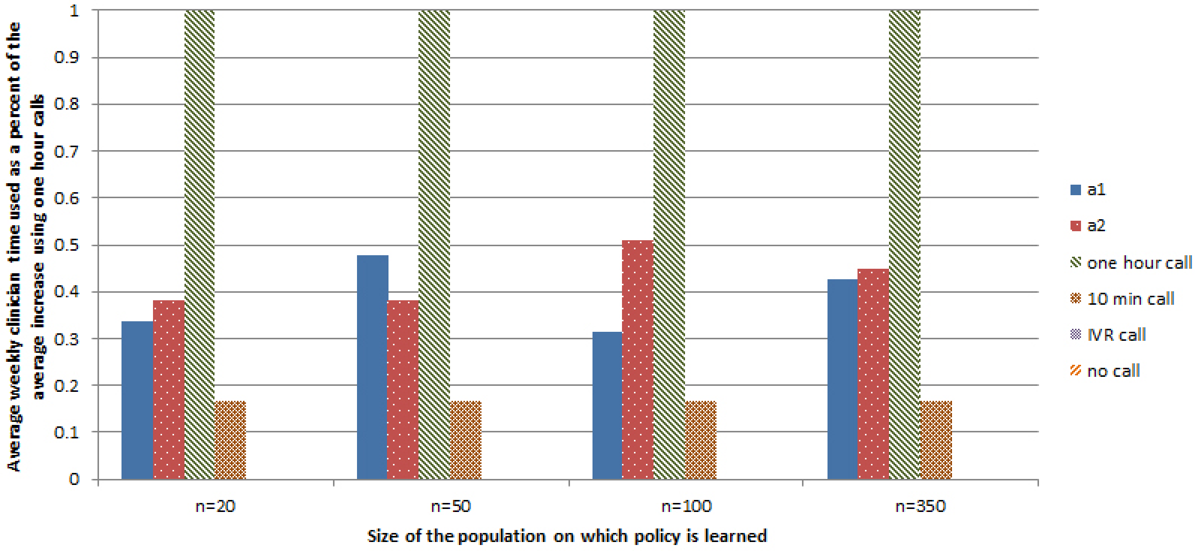

Figure 2: Average Clinician Time Used, as a Percent of the Weekly Time Consumed by Using One Hour Calls (assuming no noise)

Results Assuming Moderate Noise in Beta

As noise is introduced in patients' response to the various CBT modes, the performance of the AI-CBT program decreases somewhat, since the AI engine has an increasingly difficult time detecting what is the most efficient mode that can work for each patient in the population. This is illustrated in Figure 3, which shows that the increase in step counts is lower regardless of the size of the patient sample in the learning phase, relative to Figure 1 (i.e., without any noise). However, we also see the same general pattern comparing Action Choices #1 and #2, i.e., that Action Choice #2 typically yields a greater increase in step counts than #1, but requires more clinician time. ***Even with a moderate amount of noise, the AI engine using Action Choices #2 to learn CBT modes, is able to achieve over 91% of the efficiency of always using the one hour CBT counselor call using the policy learned on a population of 100 patients in phase one.***

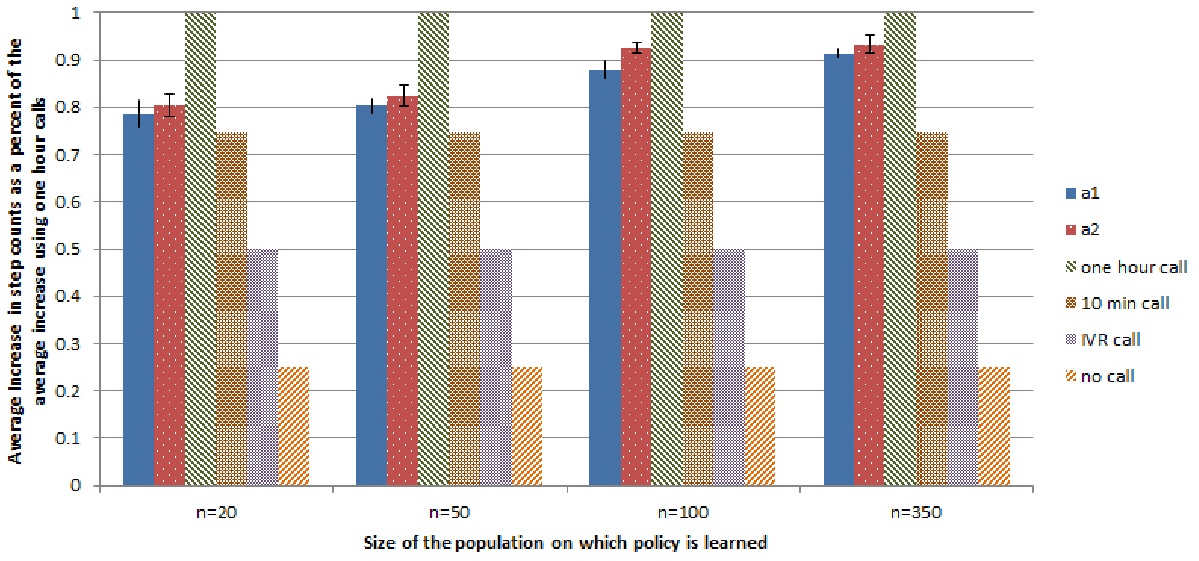

Figure 3: Average Increase in Step Counts as a Percent of the Expected Average Increase Using One Hour Calls (assuming moderate noise)

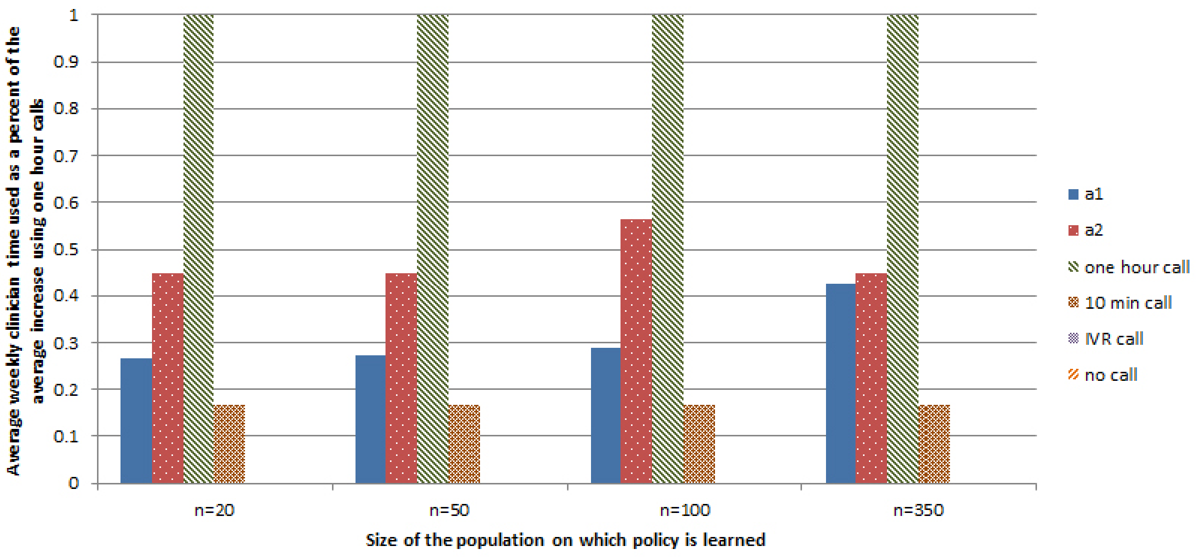

Figure 4: Average Clinician Time Used, as a Percent of the Weekly Time Consumed by Using One Hour Calls (assuming moderate noise)

Results Assuming a Large Amount of Noise in Beta

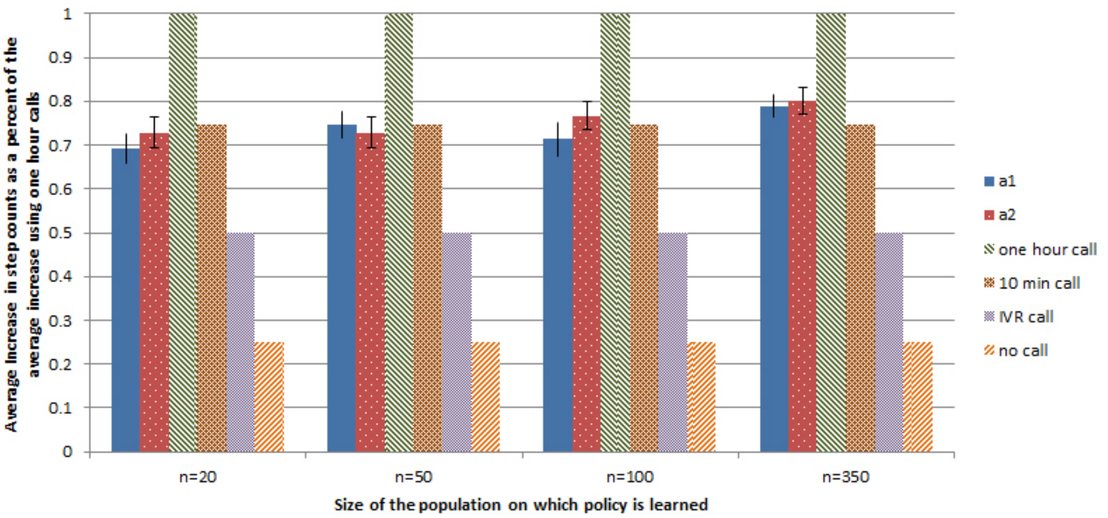

Figure 5: Average Increase in Step Counts as a Percent of the Expected Average Increase Using One Hour Calls (assuming a large amount of noise)

With a large amount of noise, we see that with a population of 100 patients in phase one, the agent using Action Choice #2 is able to achieve a roughly 78% increase in step counts relative to 10 sessions of hour long CBT. We also see that the AI engine using Action Choice #2 still in general out performs Action Choice #1.

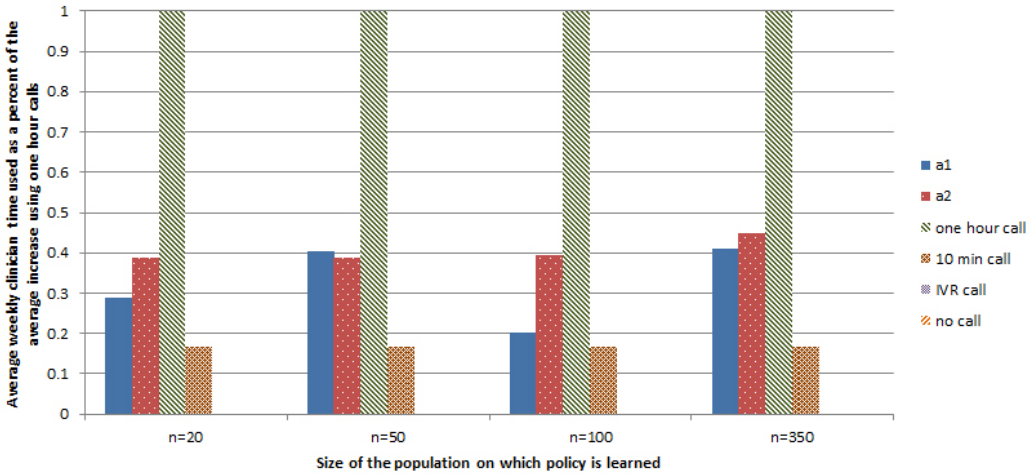

Figure 6 Average Clinician Time used, as a percent of the weekly time consumed by using one hour calls (assuming a large amount of noise)

Conclusions

These simulations suggest that, given reasonable assumptions regarding factors including: the average expected impact of a given week of CBT exposure, the proportion of patients in the population that will be responsive to various modes of service delivery, and the size of the sample, AI-CBT should be able to out-perform standard 10-sessions of CBT counseling in terms of saved clinician time, while attaining average improvements in pain-related functioning that are similar to those achieved using standard VA approaches.
