# Supplementary material for: Patient-Centered Pain Care Using Artificial Intelligence and Mobile Health Tools: Protocol for a Randomized Study Funded by the US Department of Veterans Affairs Health Services Research and Development Program
Source: JMIR Res Protoc. 2016 Apr 7;5(2):e53. doi: 10.2196/resprot.4995 (PMC4856067; doi:10.2196/resprot.4995)
Supplement: Multimedia Appendix 2 [file resprot_v5i2e53_app2.pdf]

DEPARTMENT OF VETERANS AFFAIRS
Health Services Research & Development Service
810 Vermont Ave, NW
Washington, DC 20420

July 15, 2015 In Reply Refer To: 10P9H

Director (00/151)
VA Medical Center
2215 Fuller Road
Ann Arbor, MI 48105

SUBJECT: Funding Notification
Health Services Research and Development Service
Project #: IIR 13-350
eRA application number: I01 HX001460
Title: Patient-Centered Pain Care Using Artificial Intelligence and Mobile Health Tools
Principal Investigator: John Piette, Ph.D. (Corresponding PI)
Alicia Heapy, Ph.D. (Multiple PI)

1. **Funding Decision**. I am pleased to notify you that the Health Services Research and Development Service (HSR&D) has approved funding for the subject project. Acceptance of this funding acknowledges agreement to comply with VA policies regarding intellectual property disclosure obligations and ownership rights resulting from this work. Funding will begin on **July 1, 2015**.

2. **Budget Information**. We will ask the Office of the Chief Financial Officer, Allocation Control Service, to transfer Program 824 funds to your facility for **FY15** with plans for continued support through **June 30, 2019** as detailed in the enclosed budget documents (**Enclosure 1**). Support beyond the current fiscal year is contingent upon the availability of HSR&D funds and satisfactory progress of the research. The project budget will be transmitted electronically to the Research and Development Computer Center (RDCC) office for distribution to the field.

   - **Unfilled to-be-named (TBN) positions.** When funds are required for previously unfilled positions, please e-mail Mary Jones at mary.jones@va.gov with a copy to the Scientific Portfolio Manager. The following information is needed: amount of budgeted funds needed and number of people to be hired.

3. **Reporting Requirements**. HSR&D requires three types of regular reports for every research project: annual progress report (abstract); copies of all publications based on the HSR&D-funded work; and a final report. In addition, ORD (and HSR&D) requires quad charts to be submitted for all funded studies. Quad charts must be included in all yearly annual progress reports as well as the final report. Approval of future HSR&D funding is contingent on the investigator's adherence to these critical requirements. For additional information and details regarding investigator reporting requirements, please consult your local R&D office.

   a. Annual Abstract. An annual project abstract and quad chart are due on the project funding anniversary date of each project year. Current HSR&D guidance regarding content, format, and

the process for submitting Project Abstracts is available on HSR&D's Website: http://www.hsrd.research.va.gov/funding/reporting_guidelines.cfm.

b. Publication Transmittal. Investigators are required to promptly notify HSR&D of all publications resulting from HSR&D-funded research. Submit your notification as soon as it is accepted for publication, by following the steps below:

- Go to the PubTracker Website by copying and pasting the following URL into your browser: http://vaww.pubtracker.research.va.gov/PubTracker/default.cfm (Access restricted to VA Intranet using Internet Explorer)
- Select the appropriate submission type from the "New Pre-pub Notification" and fill in the form (Be sure to upload a copy of the complete accepted article or presentation abstract).

c. Final Report. A Final Report (conforming to current HSR&D instructions) and quad chart are required at the conclusion of the funding period. Final Report instructions are available on HSR&D Website: http://www.hsrd.research.va.gov/funding/final_reports.cfm.

4. **Modification Request Policy**. Any significant modification in the approved research plan or budget requires a written request, for review and approval by HSR&D. **Approval of requests for modifications will be based on compelling justification, e.g., for situations beyond the control of the Principal Investigator that affect completion of a project as approved.** Please contact your local Research Office for details.

5. **VA Acknowledgment**. Each publication, press release or other document that cites results from VA-supported research must include an acknowledgment of VA support using the eRA application number, such as "The project described was supported by (type of award, e.g., Merit Review, Career Development Award, Pilot Project) Award Number I01 HX001460 from the United States (U.S.) Department of Veterans Affairs Health Services Research & Development Service of the VA Office of Research and Development." When the work was solely funded by VA, authors must list their VA affiliation first. When the author also holds a faculty appointment, the academic title and school also may be acknowledged. All publications should include a disclaimer similar to this statement: "The views expressed in this article are those of the author(s) and do not necessarily represent the views of the Department of Veterans Affairs."

6. **Communications**. You may direct general questions regarding this project to John P. Holden, Ph.D., Scientific Portfolio Manager, at 202-443-5735 or John.Holden3@va.gov for questions regarding the distribution of project funds, your administrative officer should contact Mary Jones at 202-443-5628 or Mary.Jones@va.gov. If, while conducting this study, you encounter any research barriers that HSR&D should be notified about and/or may assist in resolving, they should be forwarded to HSR&D by the COIN AO or Research AO via the following link: http://vaww.hsrd.research.va.gov/research-barriers/. Please include the project number (above) in any communication concerning this project. Please be reminded that all communication regarding this project should go through the local research office. The principal investigator is responsible for relating all communications from VA Central Office to any co-investigators, as necessary.

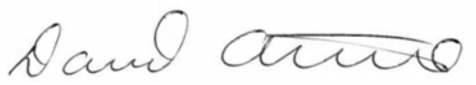

David Atkins, M.D., M.P.H.
Director, Health Services Research
and Development Service

Enclosure: Fiduciary Responsibility
Budget sheet

cc: John Piette, Ph.D. (Corresponding PI)
Alicia Heapy, Ph.D. (Multiple PI)
ACOS/R&D
COIN Director, Ann Arbor, MI
ORD Finance
Project File

Fiduciary Responsibility

It is the fiduciary responsibility of each PI, working with the local ACOS/R&D staff, to monitor the status of funding expenditures and ensure that funds are spent as described on the project budget. Funds allocated in a given fiscal year must be spent by the end of that fiscal year. In rare circumstances, CO will consider written requests to “pull back” funds but cannot guarantee that they will be returned in ensuing fiscal years. Requests to “pull back” funds must be submitted by the AO at the local R&D office to CO. Requests to “pull back” funds for return in future fiscal years are more likely to be approved if they are made early in the fiscal year or if CO has been advised of a potential problem that becomes real. Requests made after June 30th are not likely to be approved, especially if CO has not received communication of a potential problem. ANY FUNDS THAT ARE NOT SPENT WILL BE WITHDRAWN.

It is the responsibility of the PI to notify the local R&D office if a move to another VA is anticipated or if you expect to leave the VA.

To help you to meet this fiduciary obligation, we offer the following recommendations:

- Request a monthly expenditure report from your local ACOS/R&D office and review it to ensure that all charges are accurate and approximately 1/12 of your proposed budget is spent monthly
- Request notification when a Transfer Disbursing Authority (TDA) for your project has been received on station
- Communicate with your local R&D office **as soon as you are aware** of a personnel problem such as:
    - Inability to spend all of the funds allocated to you for salary due to a project member leaving or out on extended leave (sick leave or family leave)
    - Inability to spend all of the funds allocated to you for salary due to a delay in recruiting or hiring project staff
- Communicate with your local R&D office if you will be unable to recruit an adequate number of subjects (and this will delay completion of the study)
- Communicate with your local R&D office if you are unable to obtain access to datasets that are necessary for the completion of your study or if there are problems with the data sets which will delay completion of the study
- Communicate with your local R&D office if there are problems spending IT funds. Check the status of IT requests to ensure that they have been entered into the request and approval system
- Ask your local ACOS/R&D office to include you on the “cc” line of any communication of problems/issues to CO to ensure that the communication you have initiated is transmitted.

If the project is a multi-site study, it is the responsibility of the PI to monitor expenditures at all sites, and communicate potential problems to his/her own local R&D office.

Once HSR&D funding is initiated, investigators must obtain formal approval from the Director, HSR&D, for any significant change in the approved project research plan, objectives, methods, budget, time, key personnel, or site(s). **Approval of requests for modifications will be based on compelling justification, e.g., for situations beyond the control of the Principal Investigator that affect completion of a project as approved.**

All requests for project modifications must be submitted by the medical center Director, through the ACOS for R&D and the Center Director (if applicable) to the Director, HSR&D. To permit careful review, all modification requests must be submitted as soon as the need becomes apparent and, in all cases, at least 3 months prior to the effective date of the proposed change. Justification for the requested

modification must be clear, detailed, and contain appropriate supporting documentation, including revised budgets, timelines, letters of support, etc., as applicable. If additional information is required, the Principal Investigator will have 30 days from the date of communication to respond to the request; if this deadline is not met, the request may be disapproved. Unusual or extraordinary circumstances that preclude a response by the deadline must be discussed with the Scientific Program Manager for the project.

Current instructions on required format and content for requesting Project Modifications can be found at HSR&D's website: http://www.hsrd.research.va.gov.

Department of Veterans Affairs		MERIT REVIEW BOARD SUMMARY STATEMENT		
1. PROJECT ID<br>IIR 13-350	2. APPLICATION NO<br>824-MR-II-27246	3. REVIEW GROUP<br>Merit Review	4. REVIEW DATE<br>W 2014/05	5. FACILITY NO<br>506
6. LOCATION HEALTH CARE FACILITY (VAMC, OPC, CITY, STATE)<br>Ann Arbor, MI			7. CID#<br>JPIETTE	8. DATE OF LAST M.R.
9. INVESTIGATOR (Last Name, First Name, M.I.)<br>Piette, John		DEGREE(S)<br>Ph.D.		TELEPHONE NO.<br>734-930-5620
Remarks:				
10. Project Title<br>Patient-Centered Pain Care Using Artificial Intelligence and Mobile Health Tools			Start Date:<br>Jul 1, 2015	End Date:<br>Jun 30, 2019

11. AMOUNT REQUESTED EACH YEAR

1st	2nd	3rd	4th	5th	TOTAL

12. VA EMPLOYMENT	13. VA SALARY SOURCE		14. TYPE PROGRAM
☑ Full Time	☐ Research CC103	☐ Medical Research (821)	☐ New
☐ Part Time	☐ Research CC104	☑ HSR&D (824)	☐ Ongoing
☐ Consulting	☐ Research CC105	☐ Rehab R&D (822)	☐ Supplement
☐ Contract	☐ Research CC110	☐ Coop Studies (825)	☐ Type II
☐ WOC	☐ Career Dev. CC108	☐ VA Other Than R&D	☐ NO. Projects In Program

15. PROGRAM: 824 Health Services

16. Primary Research Program Area	Primary Specialty Area
Health Services	Epidemiology

17a. VA Hospital Service	18a. Academic Rank, Affiliation
HSR&D	Associate Professor, University of Michigan
**17b. VA Hospital Section**	**18b. Department & Section**
General Medicine	Internal Medicine, Medicine

Recomendation	Scores	Duration	Start Date	End Date
			07/01/2015	06/30/2019

134 HSR&D

FUNDS NOT SUBJECT TO PRIORITY REDUCTION					
FY	Salary	Equipment	Travel	All Other	Total
2015	$ 9,902	$ 0	$ 0	$ 500	$ 10,402
2016	$ 111,382	$ 0	$ 2,400	$ 150,550	$ 264,332
2017	$ 119,726	$ 0	$ 0	$ 57,080	$ 176,806
2018	$ 120,924	$ 0	$ 0	$ 39,080	$ 160,004
2019	$ 41,263	$ 0	$ 0	$ 29,600	$ 70,863

Department of Veterans Affairs	MERIT REVIEW BOARD SUMMARY STATEMENT			
1. PROJECT ID<br>IIR 13-350	2. APPLICATION NO<br>824-MR-II-27246	3. REVIEW GROUP<br>Merit Review	4. REVIEW DATE<br>W 2014/05	5. FACILITY NO<br>689
6. LOCATION HEALTH CARE FACILITY (VAMC, OPC, CITY, STATE)<br>West Haven, CT			7. CID#<br>AHEAPY	8. DATE OF LAST M.R.
9. INVESTIGATOR (Last Name, First Name, M.I.)<br>Heapy, Alicia	DEGREE(S)<br>Ph.D.			TELEPHONE NO.<br>203-932-5711 2299
Remarks:				
10. Project Title<br>Patient-Centered Pain Care Using Artificial Intelligence and Mobile Health Tools			Start Date:<br>Jul 1, 2015	End Date:<br>Jun 30, 2019
11. AMOUNT REQUESTED EACH YEAR<br>1st 2nd 3rd 4th 5th TOTAL				

12. VA EMPLOYMENT	13. VA SALARY SOURCE		14. TYPE PROGRAM
☑ Full Time	☐ Research CC103	☐ Medical Research (821)	☐ New
☐ Part Time	☐ Research CC104	☐ HSR&D (824)	☐ Ongoing
☐ Consulting	☐ Research CC105	☐ Rehab R&D (822)	☐ Supplement
☐ Contract	☐ Research CC110	☐ Coop Studies (825)	☐ Type II
☐ WOC	☐ Career Dev. CC108	☑ VA Other Than R&D	☐ NO. Projects In Program

15. PROGRAM: 824 Health Services

16. Primary Research Program Area	Primary Specialty Area
Behavioral Sciences	Psychology

17a. VA Hospital Service	18a. Academic Rank, Affiliation
Medical Research	Other, Yale University School of Medicine
**17b. VA Hospital Section**	**18b. Department & Section**
Psychology	Psychiatry, Psychiatry

Recomendation	Scores	Duration	Start Date	End Date
			07/01/2015	06/30/2019

134 HSR&D

FUNDS NOT SUBJECT TO PRIORITY REDUCTION					
FY	Salary	Equipment	Travel	All Other	Total
2015	$ 8,878	$ 0	$ 0	$ 500	$ 9,378
2016	$ 99,858	$ 0	$ 1,200	$ 8,030	$ 109,088
2017	$ 134,328	$ 0	$ 0	$ 3,830	$ 138,158
2018	$ 95,559	$ 0	$ 0	$ 3,230	$ 98,789
2019	$ 61,817	$ 0	$ 0	$ 0	$ 61,817
